# The effect of mindfulness training interventions on free throw performance in basketball players in stressful situations

**DOI:** 10.3389/fpsyg.2025.1647698

**Published:** 2025-09-17

**Authors:** Xiaotao Wang, Ziyun Zhang

**Affiliations:** ^1^Hainan Police College, Haikou, China; ^2^School of Life and Health, Huzhou College, Huzhou, China

**Keywords:** mindfulness training, basketball, free throws, stress, mental toughness

## Abstract

**Objective:**

To explore the effect of mindfulness training interventions on the free throw performance of basketball players in stressful situations.

**Methods:**

A 2 (high stress, low stress) × 2 (mindfulness training, placebo) factorial design was used. Seventy-nine male college students majoring in basketball from universities, who had experience in provincial-level or above basketball competitions, were recruited and randomly divided into 4 groups. The mindfulness group received 8 weeks of mindfulness training, with 2 sessions per week and each session lasting 90 min, while the placebo group watched biographical videos of basketball figures during the same period. The Five Facets Mindfulness Questionnaire, the Chinese version of the Connor-Davidson Resilience Scale, and a specific free throw scoring method were used to measure mindfulness levels, mental toughness, and free throw performance before and after the intervention. Based on SPSS 25.0 software, statistical analyses were conducted using one-way analysis of variance, the linear response model of the Generalized Linear Model, and Pearson correlation analysis.

**Results:**

Mindfulness training significantly improved the mindfulness levels, mental toughness, and free throw performance of athletes (*p* < 0.05). There were significant positive correlations among mindfulness levels, mental toughness, and free throw performance (*p* < 0.05).

**Conclusion:**

Mindfulness training is helpful for improving the free throw performance of basketball players in stressful situations.

## 1 Introduction

In the field of competitive sports, athletic performance under stress is not only a key factor determining the outcome of a competition, but also a core challenge for the sustainable development of an athlete’s career. From a neurobiological perspective, when athletes face high-stress situations, the over activation of the sympathetic nervous system leads to elevated cortisol levels, which in turn interferes with the fine neuromuscular coordination required for the execution of motor skills (Britton et al., 2019; Lee et al., 2023; Li et al., 2019). This physiological stress response is referred to in sports psychology as the “choking under stress” phenomenon (Mesagno et al., 2015), which can significantly reduce the stability of athletic performance (Hill et al., 2010).

Free throws refer to the opportunity to shoot from a fixed point on the free-throw line in basketball games, which is granted due to a player being fouled or when a violation occurs. Free throws are the only scoring method in basketball games that are not directly interfered with by opponents and are non-competitive (Botsi et al., 2024). Although free throws may seem the easiest, players often need to overcome tangible or intangible stress such as the audience, self-threats, and performance expectations. This can lead to technical deformation and disruption of the fluidity of movements during crucial moments in important games, resulting in failed free throws (Butler and Baumeister, 1998; Wang, 2003). Zhang (2018) found that the performance of high-level basketball players in five-point shooting under high-stress situations significantly decreased. Therefore, how to help athletes cope with high-stress situations has gradually become a topic of interest for researchers.

Mindfulness training, as a psychological intervention, has been widely applied to improve athletes’ psychological quality and sports performance (Aherne et al., 2011; Röthlin et al., 2016). Mindfulness is a state of consciousness characterized by focused, non-judgmental awareness of the present moment (Kabat-Zinn et al., 1985), and maintaining this trait is of great importance in competitive sports. Mindfulness training aims to help individuals improve their attention, emotional regulation, and stress management skills (De Vibe et al., 2013; Verhaeghen, 2021; Zhang et al., 2019). In competitive sports, mindfulness training is also often used to enhance athletes’ focus, reduce anxiety and tension, thereby improving sports performance, and has achieved positive results (Baltzell and Akhtar, 2014; De Petrillo et al., 2009; Goodman et al., 2014). Studies on the intervention of mindfulness training on basketball free throw performance (Shi et al., 2018; Wang, 2020) have shown that mindfulness training can increase the level of mindfulness in basketball players, and improve their free throw performance. However, related studies (Chen et al., 2022; Wolch et al., 2021) show that mindfulness training can reduce the anxiety level of basketball players, but it does not improve free throw performance under stressful situations.

The reasons for the differences between studies may include the following points. Firstly, previous studies (Shi et al., 2018; Wang, 2020) did not fully consider the ecological validity of free-throw shooting, all of which were conducted in training settings, while testing under stressful conditions may yield inconsistent results. Secondly, studies on the intervention of mindfulness training on basketball players’ free throw performance under stressful conditions (Chen et al., 2022; Wolch et al., 2021) all used one-time interventions, and shorter intervention periods may have relatively limited effects. The limited effectiveness of shorter mindfulness interventions mainly stems from the following three reasons. Firstly, short-term interventions may struggle to form stable neural circuits, making it difficult for their effects to persist. Secondly, mindfulness is essentially a psychological skill, and beginners find it hard to grasp its core techniques within a short period, which naturally prevents the translation of such interventions into actual results. Finally, the automatic responses under stress are long-formed psychological habits; short-term interventions can only temporarily break these habits but fail to establish new coping patterns. Once individuals are away from the intervention environment, the old patterns are likely to reappear.

In the field of competitive sports, mental toughness is an important psychological quality for athletes to cope with competitive stress and achieve excellent performance (Gucciardi, 2017). Mental toughness is the psychological ability of individuals to adapt effectively, return to normalcy, and potentially gain growth when facing adversity, stress, or trauma (Gucciardi, 2017). Relevant studies (Khan et al., 2017; Schneider and Stier, 2008) have shown that athletes with high mental toughness can better regulate their emotions and cognition when facing competitive stress, maintain calmness and focus, and better cope with the potential “choking” phenomenon in competitions, thus performing at their best. The so-called Choking is a phenomenon where, in high-stress situations, an individual’s performance suddenly declines or even results in errors because psychological stress interferes with the automatic execution of well-practiced skills. Relevant studies (Ajilchi et al., 2019, 2022; Wang et al., 2021) have confirmed that mindfulness training can effectively enhance participants’ mental toughness. Ajilchi et al. (2022) found that mindfulness training can improve athletes’ mental toughness, mental health, and emotional intelligence; Wang et al. (2021) found that mindfulness training increased female college students’ mindfulness and mental toughness during endurance exercise, and reduced their perceived fatigue during endurance exercise. In basketball, Ajilchi et al. (2019), studying amateur basketball players, found that mindfulness training improved mindfulness levels and overall mental toughness, which greatly helped in enhancing their athletic performance.

Based on this, the aim of this study was to investigate the effect of mindfulness training interventions on free throw performance in basketball players in stressful situations. Through this study, it enriches the application research of mindfulness training in the field of competitive sports and provides a new perspective and method for the psychological training of basketball players.

## 2 Methods

### 2.1 Participants

This study is an exploratory research with a two-factor mixed design, and in principle, sample size calculation is not required. However, to ensure the effectiveness of mindfulness training intervention on free-throw performance, this study extracted the effect size of mindfulness training on athletes’ sports performance as 0.82 based on the systematic review and meta-analysis by Zhao (2023). Using G • Power 3.1 software, with the alpha error rate (significance level) set to 0.05 and the power (statistical power) set to 0.80, the calculation showed that a total of 50 participants were needed. Specifically, the mindfulness training group and the placebo group require 25 participants each. Due to the exploratory nature of the study design and objective constraints in sample recruitment, this study recruited 79 male college students majoring in basketball from a university in Hainan Province, China, to participate in the experiment. The average age of the participants was 21.95 ± 2.19 years old. These college students all indicated that they had participated in basketball competitions at the provincial level (where “provincial level” refers to competitions organized by the government of a first-level administrative region in China) or above, with training years ranging from 8 to 17 years, training three times a week, and each training session lasting 90 min. In addition, all participants reported that they had not received any similar mindfulness training prior to participating in the mindfulness training of this study. This study was conducted in accordance with the principles of the Helsinki Declaration. The study was approved by the Research Ethics Committee of Hainan Police College, and all participants provided informed consent.

### 2.2 Design

This study employs a 2 (high stress and low stress) × 2 (mindfulness and placebo) factorial design. Initially, participants were divided into high-stress and low-stress groups using the random number method. Subsequently, within the high-stress group, participants were further divided into a mindfulness group and a placebo group, and within the low-stress group, participants were divided into a mindfulness group and a placebo group using the same method. The stress scenarios were set up using audience interference and camera interference. The mindfulness group received an 8-week mindfulness training intervention, while the placebo group received an 8-week video intervention. A single-blind strategy was implemented for the training to prevent participants from experiencing the Hawthorne Effect. In addition, the outcome variables in this study include levels of mindfulness, psychological resilience, and free throw performance. In addition, this study conducted free throw tests under both high-stress and low-stress conditions for all groups to ensure consistency among them. Before and after the intervention, the high-stress group underwent free throw tests under high-stress conditions, and the low-stress group underwent free throw tests under low-stress conditions.

### 2.3 Procedure

This study conducted mindfulness training for participants in the laboratory of Hainan Police College. The mindfulness training intervention period lasted for 8 weeks, with two sessions per week held on Tuesday and Friday evenings from 8:00 PM to 9:30 PM, each intervention lasting 90 min. This study employed the Mindfulness-Acceptance-Insight-Commitment (MAIC) training program to train the mindfulness group (Su et al., 2019). The core reasons for this study to select MAIC include the following two aspects. Firstly, MAIC is specifically designed for Chinese athletes and meets the needs of cultural adaptability. The MAIC program, proposed by Si et al. (2014), has its core advantage in localized design. MAIC fully takes into account the way of thinking, training habits and cultural psychological characteristics of Chinese athletes, making it easier for athletes to understand and accept. In addition, this program has been widely applied in various sports such as shooting, tennis, and synchronized swimming in mainland China and Hong Kong (Su et al., 2019). Empirical data show that it has high compatibility with psychological interventions for athletes in Chinese cultural backgrounds and can effectively reduce the discount of intervention effects caused by cultural differences (Bu et al., 2020; Feng and Si, 2015). This is crucial for the design of this study, which takes Chinese college basketball players as samples. (2) MAIC focuses on improving sports performance and is highly matched with the research objectives. MAIC clearly reflects the orientation towards sports scenarios. Relevant studies (Bu et al., 2020; Feng and Si, 2015; Su et al., 2019) have confirmed that MAIC can significantly improve athletes’ concentration, emotional regulation ability and sports performance, which are the core influencing factors of free-throw performance under stress concerned in this study.

Based on the Athlete Mindfulness Manual compiled by Si et al. (2014), the study implemented interventions for the mindfulness group. During each 90-min mindfulness training session, first, the researcher conducted approximately 30 min of explanations on mindfulness training content, helping athletes become familiar with the process of mindfulness training to better carry out the practice. Secondly, the researcher guided approximately 60 min of mindfulness audio follow-along exercises, which included mindfulness breathing exercises, concentration exercises, body scan exercises, mindful fruit eating exercises, slow- motion water drinking exercises, mindful walking exercises, mindfulness meditation exercises, and mindfulness number exercises. In the MAIC training program adopted in this study, all selected exercises are directly related to the cultivation of core mindfulness elements, and their design logic closely focuses on improving athletes’ psychological adjustment ability and movement stability in stressful situations. Specifically, the pertinence of each exercise is as follows:

1. Mindful breathing exercise: As a basic introductory exercise, its core goal is to strengthen the mindfulness abilities in the dimensions of “observing” and “non-judging.” By guiding athletes to focus on the natural rhythm of breathing (such as the flow of air through the nostrils and the rise and fall of the abdomen), it helps them quickly bring their attention back to the present moment when distracted by random thoughts, reducing excessive worries about past mistakes or future outcomes. This exercise lays the foundation for maintaining concentration in subsequent complex scenarios, and is particularly suitable for stages of action execution that require a high degree of concentration, such as free throws.
2. Concentration exercise: By focusing on a single object, it deliberately trains athletes’ ability to resist environmental interference, directly corresponding to the “acting with awareness” dimension. In basketball free-throw scenarios, athletes often face external stimuli such as audience noise and opponent interference. This exercise can enhance their sustained attention to the target (the basket) and reduce the disruption of irrelevant information to the coherence of movements.
3. Body scan exercise: It requires athletes to perceive physical sensations part by part (from toes to the top of the head), focusing on cultivating “observing” and “describing” abilities. For free throws, accurate physical perception (such as wrist force and knee bending angle) is crucial for standardizing movements. This exercise can help athletes identify physical tension signals under stress (such as stiff shoulders and neck) and restore the best state through active adjustment.
4. Mindful fruit eating/slow-motion drinking exercise: By slowing down daily actions and carefully perceiving sensory experiences (such as the texture of fruits and the feel of water flowing through the throat), it strengthens the “acting with awareness” and “non-reacting” dimensions. Such exercises simulate the need to “break down movement steps” during free throws, prompting athletes to get rid of the automatic inertia of movements and avoid movement deformities caused by stress (such as hasty shots).
5. Mindful walking exercise: Maintaining awareness of foot landing and body weight transfer during movement, combining the state of mindfulness with dynamic actions, is closer to the actual scene of basketball. Its role is to train athletes to maintain concentration during physical activities, preventing distractions during movement (such as outside movements) from affecting subsequent free-throw preparations.
6. Mindful meditation exercise: Through open awareness (such as accepting all thoughts without getting involved), it deepens the abilities of “non-judging” and “non-reacting.” This is directly related to emotional management under stress - when athletes have self-doubt due to mistakes, this exercise can help them view emotions with a neutral attitude and prevent the spread of negative thoughts from affecting the next free throw.
7. Mindful number exercise: It trains the stability of attention through counting down numbers or memorizing number sequences, and introduces slight interference (such as occasional sound prompts) to simulate sudden stress in competitions. This exercise specifically improves athletes’ ability to quickly recover after their attention is briefly interrupted, which is highly consistent with the scenario of dealing with sudden cheers from the audience during free throws.

These exercises are not randomly selected; instead, they gradually cover the five core dimensions of mindfulness (observing, describing, acting with awareness, non-judging, and non-reacting) through step-by-step training. They are also closely integrated with the movement characteristics of basketball free throws and the needs of stressful scenarios, ultimately achieving the transfer from basic psychological skills to specific performance. One researcher led each 90-min intervention session on site, recorded participants’ attendance through a sign-in sheet, and ensured that participants received the normal intervention. During the intervention, the researcher made records by observing the participants’ concentration on site (such as whether they followed the audio instructions to complete the actions and whether there were frequent distractions). In addition, the non-mindfulness control group also engaged in an 8-week program with two sessions per week, each lasting 90 min, watching videos that featured biographical stories of legendary basketball figures.

### 2.4 Measures

#### 2.4.1 Five facets mindfulness questionnaire (FFMQ)

This study employed the Five Facets Mindfulness Questionnaire (FFMQ) to measure the mindfulness levels of basketball athletes. The FFMQ was translated and revised from the English version by Deng et al. (2011). The questionnaire consists of 39 items, with 19 of them being reverse-scored items. It uses a 5-point Likert scale ranging from 1 (not at all true) to 5 (completely true). The scale encompasses five dimensions: observing, describing, acting with awareness, non-judging, and non- reacting. Specifically, the Observing, Describing, and Acting with Awareness dimensions each contain 8 items, while the non-judging dimension and non-reacting dimension has 7 items. For example, an item for the observing dimension is “I observe my emotions without getting lost in them”; an item for the describing dimension is “I always tend to describe my experiences in words”; an item for the acting with awareness dimension is “When I walk, I pay attention to the sensations in different parts of my body as I move”; an item for the non-judging dimension is “I judge my thoughts as good or bad”; and an item for the non-reacting dimension is “I feel my emotions and feelings, but I do not have to react to them.” Higher scores on each dimension indicate stronger mindfulness abilities in that area; the higher the total score across all dimensions, the greater the overall mindfulness ability. Deng et al. (2011) confirmed that the Chinese version of the FFMQ possesses good psychometric properties (Cronbach’s *α* = 0.86) and is an effective tool for assessing mindfulness levels.

#### 2.4.2 Connor-Davidson resilience scale-Chinese version (CDRISC)

This study employed the CDRISC revised by Yu and Zhang (2007) to assess participants’ mental toughness. The scale consists of a total of 25 items, each scored on a 5-point Likert scale, where 1 to 5 indicates a range from completely disagree to completely agree. The scale is divided into three dimensions: resilience, self-improvement, and optimism. For example, an item for the resilience dimension is “despite the obstacles, I believe I can achieve my goals”; an item for the self-improvement dimension is “I have become stronger because I have gone through hardships”; and an item for the optimism dimension is “When facing problems, I try to see the humorous side of things.” Higher scores on each dimension indicate greater capacity in that area. The higher the total score across all dimensions, the higher the overall level of mental toughness. The scale has an internal consistency coefficient of 0.91, indicating good reliability.

#### 2.4.3 Free throw performance

This study tested the free throw performance of basketball players under high-stress and low-stress situations. High-stress situations were simulated through the presence of an audience and cameras. According to social facilitation theory, the presence of others can enhance performance on simple or well-learned tasks but can be detrimental to the performance of complex or non-automated tasks (Baumeister et al., 1985). The expectations of the audience may also affect an individual’s perception of stress, with audience expectations potentially acting as a mediating factor. Baumeister et al. (1985) found that audience expectations can reduce performance, as performers, in an attempt to meet high expectations, increase their own stress, which in turn affects their athletic performance. Therefore, this study, with reference to Zhou (2023), recruited 20 spectators to be distributed on both sides of the free-throw area to create distractions. Spectators could implement disturbances in various forms, such as whistling, using clappers, and cheering sticks. Secondly, with the commercialization of sports, cameras have increasingly appeared in competitions. Research by Wang et al. (2004) has shown that cameras, similar in essence to the presence of an audience, increase an athlete’s self-awareness and perceived stress, thus cameras can also serve as a means of stress manipulation. In this study, players were informed that they would be filmed throughout, but in reality, only three cameras were set up at different positions on the court and kept on, without actual recording. Relevant studies (Baumeister et al., 1985; Wang et al., 2004) have confirmed that these two strategies have high ecological validity and can induce a higher level of psychological stress in basketball players. In the low-stress condition, there were no such situational elements as spectators and cameras that could induce psychological stress in players.

This study employed the scoring method system developed by Pates et al. (2002) to evaluate the quality and performance of each free throw shot, which is more sensitive in reflecting the performance of free throws than simply counting whether the shot goes in or the shooting percentage. The scoring method is as follows: a shot that goes in without touching the backboard or the rim (a swish), scores 5 points; a shot that touches the rim first before going in (including shots that touch the rim and then the backboard), scores 4 points; a shot that touches the backboard first before going in (including shots that touch the backboard and then the rim), scores 3 points; a shot that touches the rim and does not go in (including shots that touch the rim and then the backboard without going in), scores 2 points; a shot that touches the backboard and does not go in (including shots that touch the backboard and then the rim without going in), scores 1 point; a shot that does not touch anything and does not go in, scores 0 points. The free throw score is recorded by adding up the points from each person’s 10 free throw attempts, with possible scores ranging from 0 to 50.

### 2.5 Data analysis

This study employs SPSS 25.0 software for data processing and statistical analysis. Firstly, a one-way analysis of variance (ANOVA) was used for inter-group comparisons. Before conducting the ANOVA analysis, the Levene’s test was performed to test for homogeneity of variances; if the variances were homogeneous, the Least Significant Difference method was used for pairwise comparisons between groups; if the variances were not homogeneous, Tamhane’s T2 test was used for inter-group comparisons. Secondly, this study employed a univariate two-way analysis of variance (ANOVA) to analyze the intervention effect of mindfulness training. Herein, the dependent variables were mindfulness level, mental toughness, and free-throw performance; the between-participants variable was whether mindfulness training was received (mindfulness training group vs. placebo group); and the within-participants variable was stress condition (high stress vs. low stress). In the analysis, an interaction effect of “mindfulness training (mindfulness group, placebo group) × stress condition (high stress, low stress)” was constructed to examine the changes in basketball players under different stress conditions before and after mindfulness training. Additionally, the Bonferroni method was used for pairwise comparison tests, and partial *η*^2^ was adopted to represent the effect size. Finally, Pearson correlation analysis is used to explore the correlations between post-intervention mindfulness levels, mental toughness, and free throw performance. In addition, this study used the linear interpolation method to impute missing values. The significance level in this study was set at *α* = 0.05.

## 3 Results

### 3.1 Basic information of participants

The average age of the high-stress mindfulness group is (21.80 ± 2.17) years, the average age of the high-stress placebo group is (22.05 ± 2.46) years, the average age of the low-stress mindfulness group is (21.75 ± 2.12) years, and the average age of the low-stress placebo group is (22.21 ± 2.12) years. There is no significant difference between the groups (*F* = 0.185, *p* = 0.907). Additionally, there are no significant differences in the mindfulness scores, mental toughness scores, and free-throw performance under low and high-stress situations among the groups (*p* > 0.05). The results indicate that the four groups had a high degree of homogeneity at baseline, which allows for subsequent intervention experiments and statistical analysis. The intergroup comparison results of the participants’ pretest data are detailed in Table 1.

**Table 1:** Comparative between-group analysis of participant pre-test data (*M* ± *SD*).

Variables	High-stress mindfulness group (*n* = 20)	High-stress placebo group (*n* = 20)	Low-stress mindfulness group (*n* = 20)	Low-stress placebo group (*n* = 19)	*F*	*P*
Age	21.80 ± 2.17	22.05 ± 2.46	21.75 ± 2.12	22.21 ± 2.12	0.185	0.907
Observing	29.40 ± 1.93	29.50 ± 1.85	29.65 ± 2.48	29.37 ± 2.39	0.067	0.977
Describing	28.50 ± 1.91	28.90 ± 2.43	28.90 ± 2.32	28.95 ± 2.42	0.168	0.918
Acting with awareness	26.60 ± 2.11	26.75 ± 1.92	26.60 ± 1.60	27.05 ± 1.13	0.292	0.831
Non-judging	22.45 ± 1.57	22.95 ± 2.35	22.45 ± 1.50	23.21 ± 1.40	0.915	0.438
Non-reacting	23.90 ± 1.52	24.20 ± 1.54	23.70 ± 2.08	23.47 ± 1.58	0.646	0.588
Mindfulness	130.85 ± 4.88	132.30 ± 5.60	131.30 ± 4.74	132.05 ± 5.25	0.339	0.797
Resilience	48.35 ± 2.23	48.05 ± 2.78	49.35 ± 2.06	48.79 ± 2.28	1.153	0.334
Self-improvement	33.70 ± 0.98	33.20 ± 1.15	33.95 ± 1.64	33.89 ± 1.60	1.240	0.301
Optimism	15.25 ± 1.12	15.40 ± 1.14	14.80 ± 2.31	15.37 ± 1.01	0.682	0.566
Mental toughness	97.30 ± 2.45	96.65 ± 2.72	98.10 ± 3.60	98.05 ± 3.18	1.032	0.383
Free throw performance under high-stress situations	32.90 ± 1.65	32.55 ± 2.01	32.15 ± 1.69	31.79 ± 1.84	1.393	0.252
Free throw performance under low-stress situations	30.65 ± 1.27	30.50 ± 1.00	30.25 ± 1.37	30.00 ± 1.16	1.092	0.358

### 3.2 Effects of mindfulness training intervention on mindfulness levels

**Figure 1:**
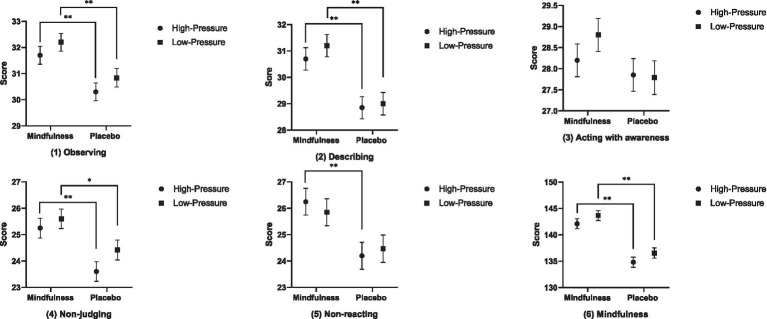
The comparison analysis results of the mindfulness levels and their dimensions for each group after intervention.

Firstly, the main effect of situation on the observing (*F* = 2.232, *p* = 0.139, *η*^2^ = 0.029), describing (*F* = 0.567, *p* = 0.454, *η*^2^ = 0.007), acting with awareness (*F* = 0.460, *p* = 0.500, *η*^2^ = 0.006), non-judging (*F* = 2.372, *p* = 0.128, *η*^2^ = 0.031), non-reacting (*F* = 0.014, *p* = 0.905, *η*^2^ = 0.000), and total mindfulness score (*F* = 2.782, *p* = 0.100, *η*^2^ = 0.036) was not significant. Secondly, the main effect of mindfulness training was significant on the observing (*F* = 15.629, *p* = 0.000, *η*^2^ = 0.172), describing (*F* = 22.001, *p* = 0.000, *η*^2^ = 0.227), non-judging (*F* = 13.843, *p* = 0.000, *η*^2^ = 0.156), non-reacting (*F* = 10.592, *p* = 0.002, *η*^2^ = 0.124), and total mindfulness score (*F* = 53.913, *p* = 0.000, *η*^2^ = 0.418), with the mindfulness group scoring significantly higher than the placebo group; however, the main effect of mindfulness training on the acting with awareness was not significant (*F* = 2.927, *p* = 0.091, *η*^2^ = 0.038). Finally, the interaction effect of situation and mindfulness training on the observing (*F* = 0.004, *p* = 0.952, *η*^2^ = 0.000), describing (*F* = 0.164, *p* = 0.686, *η*^2^ = 0.002), acting with awareness (*F* = 0.690, *p* = 0.409, *η*^2^ = 0.009), non-judging (*F* = 0.384, *p* = 0.537, *η*^2^ = 0.005), non-reacting (*F* = 0.409, *p* = 0.524, *η*^2^ = 0.005), and total mindfulness score (*F* = 0.008, *p* = 0.929, *η*^2^ = 0.000) was not significant. Nevertheless, further tests revealed that under high-stress situations, mindfulness training exerted a positive intervention effect on the observing (*F* = 8.161, *p* = 0.006, *η*^2^ = 0.098), describing (*F* = 9.302, *p* = 0.003, *η*^2^ = 0.110), non-judging (*F* = 9.542, *p* = 0.003, *η*^2^ = 0.113), non-reacting (*F* = 7.683, *p* = 0.007, *η*^2^ = 0.093), and total mindfulness score (*F* = 27.983, *p* = 0.000, *η*^2^ = 0.272), meaning the mindfulness group scored significantly higher than the placebo group in the aforementioned dimensions. Under low-stress situations, mindfulness training also demonstrated a positive intervention effect on the observing (*F* = 7.481, *p* = 0.008, *η*^2^ = 0.091), describing (*F* = 12.817, *p* = 0.001, *η*^2^ = 0.146), non-judging (*F* = 4.747, *p* = 0.032, *η*^2^ = 0.060), and total mindfulness score (*F* = 25.964, *p* = 0.000, *η*^2^ = 0.257), indicating the mindfulness group obtained significantly higher scores than the placebo group in these dimensions. The results of the comparative analysis of mindfulness levels and their respective dimensions across all groups after the intervention are presented in Figure 1.

### 3.3 Effects of mindfulness training intervention on mental toughness

**Figure 2:**
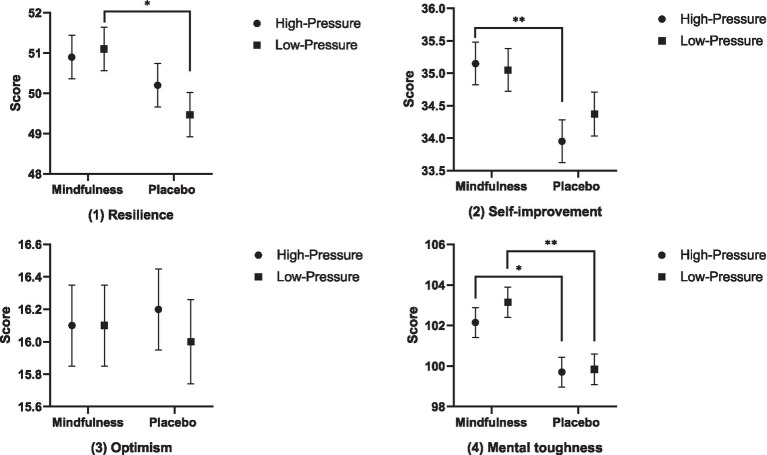
The comparison analysis results of the mental toughness and their dimensions for each group after intervention.

Firstly, the main effect of situation on the resilience (*F* = 0.225, *p* = 0.637, *η*^2^ = 0.637), self-improvement (*F* = 0.217, *p* = 0.643, *η*^2^ = 0.003), optimism (*F* = 0.147, *p* = 0.703, *η*^2^ = 0.002), and total mental toughness score (*F* = 0.565, *p* = 0.454, *η*^2^ = 0.007) was not significant. Secondly, the main effect of mindfulness training was significant on the resilience (*F* = 4.397, *p* = 0.039, *η*^2^ = 0.055), self-improvement (*F* = 7.580, *p* = 0.007, *η*^2^ = 0.092), and total mental toughness score (*F* = 14.372, *p* = 0.000, *η*^2^ = 0.161), with the mindfulness group scoring significantly higher than the placebo group; however, its main effect on the optimism was not significant (*F* = 0.000, *p* = 1.000, *η*^2^ = 0.000). Finally, the interaction effect of situation and mindfulness training on the resilience (*F* = 0.697, *p* = 0.406, *η*^2^ = 0.009), self-improvement (*F* = 0.575, *p* = 0.450, *η*^2^ = 0.008), optimism (*F* = 0.147, *p* = 0.703, *η*^2^ = 0.002), and total mental toughness score (*F* = 0.319, *p* = 0.574, *η*^2^ = 0.004) was not significant. Nevertheless, further tests showed that under high-stress situations, mindfulness training had a significant intervention effect on the self-improvement (*F* = 6.247, *p* = 0.015, *η*^2^ = 0.077) and total mental toughness score (*F* = 5.273, *p* = 0.024, *η*^2^ = 0.066), meaning the mindfulness group scored significantly higher than the placebo group; under low-stress situations, mindfulness training exerted a significant intervention effect on the resilience (*F* = 4.243, *p* = 0.043, *η*^2^ = 0.054) and total mental toughness score (*F* = 9.365, *p* = 0.003, *η*^2^ = 0.111), indicating the mindfulness group obtained significantly higher scores than the placebo group. In summary, mindfulness training can effectively enhance the mental toughness level of basketball players. The results of the comparative analysis of mental toughness and its respective dimensions across all groups after the intervention are presented in Figure 2.

### 3.4 Effects of mindfulness training intervention on free throw performance

**Figure 3:**
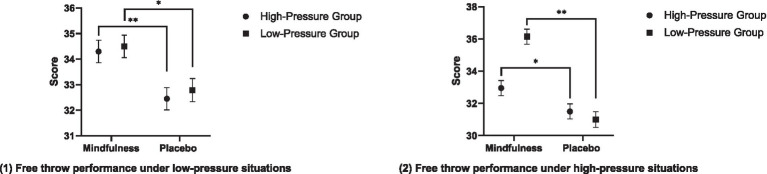
The comparison analysis results of the free throw performance and their dimensions for each group after intervention.

Firstly, under the low-stress condition, the main effect of the situation on free throw performance was not significant (*F* = 0.355, *p* = 0.553, *η*^2^ = 0.005); the main effect of mindfulness training was significant (*F* = 15.470, *p* = 0.000, *η*^2^ = 0.171), with the free throw performance of the mindfulness group being significantly better than that of the placebo group; the interaction effect between the situation and mindfulness training was not significant (*F* = 0.024, *p* = 0.878, *η*^2^ = 0.000). The pairwise comparison analysis showed that the free throw performance of the high-stress mindfulness group was significantly better than that of the high-stress placebo group, and the free throw performance of the low-stress mindfulness group was significantly better than that of the low-stress placebo group. Secondly, under the high-stress condition, the main effect of the situation on free throw performance was significant (*F* = 14.294, *p* = 0.000, *η*^2^ = 0.160), with the free throw performance of the low-stress group being higher than that of the high-stress group; the main effect of mindfulness training was significant (*F* = 32.743, *p* = 0.000, *η*^2^ = 0.304), and the free throw performance of the mindfulness group was significantly better than that of the placebo group; the interaction effect between the situation and mindfulness training was significant (*F* = 23.064, *p* = 0.000, *η*^2^ = 0.235). The pairwise comparison analysis showed that the free throw performance of the high-stress mindfulness group was significantly better than that of the high-stress placebo group, and the free throw performance of the low-stress mindfulness group was significantly better than that of the low-stress placebo group. In summary, mindfulness training can improve the free throw performance of basketball players under both low-stress and high-stress conditions. The results of the comparative analysis of free throw performance of each group after the intervention are shown in Figure 3.

### 3.5 Correlations between mindfulness, mental toughness and free throw performance

The correlation analysis results (Table 2) of basketball players’ mindfulness levels, mental toughness, and free throw performance indicate that there is a significant (*p* < 0.05) positive correlations between mindfulness levels and mental toughness (*r* = 0.225), free throw performance under high-stress situations (*r* = 0.229), and free throw performance under low- stress situations (*r* = 0.314); mental toughness also shows a significant (p < 0.05) positive correlation with free throw performance under high-stress situations (*r* = 0.252) and free throw performance under low-stress situations (*r* = 0.254). In summary, mindfulness, mental toughness, and basketball players’ free throw performance exhibit significant positive correlations.

**Table 2:** Results of correlation analysis of mindfulness, mental toughness and free throw performance.

Variables	Mindfulness	Mental toughness	Free throw performance under high-stress situations	Free throw performance under low-stress situations
Mindfulness	1.00	0.225*	0.229*	0.314**
Mental toughness	0.225*	1.00	0.252*	0.254*
Free throw performance under high-stress situations	0.229*	0.252*	1.00	0.212
Free throw performance under low-stress situations	0.314**	0.254*	0.212	1.00

*P < 0.05; **p < 0.01.

## 4 Discussion

Through an 8-week mindfulness training intervention, this study explored the impact of mindfulness training on basketball players’ free-throw performance in stressful situations. It was found that mindfulness training can significantly improve basketball players’ mindfulness levels, mental toughness, and free-throw performance in stressful situations. In summary, this study confirms that mindfulness training is an effective psychological intervention method to improve basketball players’ free-throw performance.

### 4.1 Discussion of the main results

The results of this study demonstrate that mindfulness training can enhance the mindfulness and mental toughness of basketball players, as well as their free throw performance, regardless of whether they are shooting under stressful conditions or not. The results of this study support previous research (Ajilchi et al., 2019; Wang et al., 2021; Zhou, 2023), all indicating that mindfulness intervention can improve players’ mental toughness and sports performance. Mindfulness training is a psychological training method aimed at improving self-awareness by encouraging individuals to focus on the present experience (Aránega et al., 2020). The potential reasons for the improvement in basketball players’ free throw performance due to mindfulness training are as follows:

Firstly, mindfulness training emphasizes full engagement and attention to the current activity, which can help athletes reduce distractions during free throws and concentrate on their technique and target (Wolch, 2019). Secondly, it can increase athletes’ awareness of their bodily sensations, allowing them to better perceive and adjust their physical state, which is essential for the precise body control required in free throws (Gao and Zhang, 2023). Thirdly, mindfulness training assists athletes in identifying and managing their emotions, especially in stressful situations, enabling them to stay calm, reduce anxiety, and tension, which is particularly important for the precise control needed in free throws (Gross et al., 2018; Josefsson et al., 2019). Fourthly, through mindfulness training, athletes may gain a deeper understanding and trust in their abilities (Gardner and Moore, 2004), and it also strengthens their mental toughness (Wang et al., 2021), allowing them to better cope with stress and maintain stable performance during free throws. Fifthly, in high-stress situations, athletes might be distracted by automatic thoughts that could affect their performance (Roberts et al., 2019); however, mindfulness training can help them recognize and eliminate these distractions (Wagner and Wieczorek, 2024; Wolch, 2019). Finally, mindfulness training encourages non-judgmental self-observation, which helps reduce self-criticism and the psychological burden caused by self-doubt (Amada and Shane, 2019). Even if a free throw is not successful, it can help athletes quickly recover from failure, maintain a positive attitude, and prepare for the next attempt.

In conclusion, mindfulness training can enhance the flow state of basketball players, improving their focus, emotion regulation, self-confidence, psychological resilience, and other abilities during free throws. These capabilities work together to help basketball players improve their free-throw performance in both high-pressure and low-pressure situations. Relevant studies (Chen et al., 2018; Schutte and Malouff, 2023) have also confirmed that although mindfulness and flow state are not the same concept, they are not opposites either; instead, they exhibit a dynamic relationship characterized by “foundation-promotion” and “regulation-balance.” The key prerequisites for triggering a flow state are highly concentrated attention and reduced interference from self-criticism, and mindfulness is precisely effective in targeted training of these abilities (Chen et al., 2018; Schutte and Malouff, 2023).

### 4.2 Practical implications

This study provides a scientific psychological training method, which helps coaches and basketball players to train more scientifically. The results of this study support the use of mindfulness training as a psychological training method to improve the mental toughness and free throw performance of basketball players. This method can help athletes maintain or enhance their competitive performance under stressful conditions. Mindfulness training is of great significance in resisting the “choking” phenomenon that may occur in competitions (i.e., performance decline under stress) and in maintaining stable performance during competitions. In addition, this study points out that different individuals may have different responses to mindfulness training, indicating that trainers need to consider individual differences and customize personalized training plans for different athletes. In summary, this study not only provides a scientific basis for the training of basketball players but also offers valuable references for psychological training in other sports.

### 4.3 Limitations of this study and directions for future studies

Although this study has achieved some positive results, it also has some limitations. Firstly, this study has a small sample size and weak representativeness. Although every effort was made to consider the homogeneity of participants during selection, other factors that may affect the results, such as athletes’ personality traits and sports experience, still cannot be ruled out. Secondly, during the experimental process, although every effort was made to ensure that each group conducted mindfulness courses according to the *Athlete Mindfulness Training Manual* and basketball story audio, it was inevitable that there would be differences in the effectiveness of the sessions when the principal investigator explained to each group. Thirdly, although irrelevant variables were controlled as much as possible, environmental settings such as weather and temperature could not be completely consistent, which may have had an impact on the research results. Finally, in terms of ecological validity, this study set up stress situations using audiences and cameras as in previous studies, but it is difficult to ensure that the stress felt by each subject is the same. We can only try to ensure that each subject is under the same stress situation, and there is still a gap between the stress situation set up and the competitive situation. In addition, this study has not yet used a participant-perceived stress scale to assess the participants’ stress levels. Therefore, it is not clear whether the participants were under stress and the level of stress they experienced, which may interfere with the research results.

In view of the limitations of this study, future research can be optimized and deepened in the following aspects. Firstly, expand the sample size, and enhance representativeness. Future studies should enrich sample diversity by including groups of different genders, ages, and sports levels to enhance the generalizability of research conclusions. Secondly, standardize the experimental procedures to reduce execution biases. To minimize the impact of differences in the explanations given by the principal investigators on the intervention effects, future studies can conduct unified training and standardized assessments for the principal investigators to ensure that they carry out guidance strictly in accordance with the preset scripts. Furthermore, a third-party supervision mechanism can be introduced, where the entire experimental process is recorded through audio and video, and the consistency of intervention implementation is checked afterwards. Thirdly, strictly control environmental variables to reduce irrelevant interference. Future research should be conducted in indoor laboratories with constant temperature and humidity as much as possible to reduce fluctuations in natural environmental factors such as weather and temperature. If it is necessary to carry out the experiments outdoors, the environmental parameters of each experiment should be recorded in detail, and the correlation between these parameters and the results should be tested in data analysis to clarify whether they constitute interference. Finally, optimize the design of stress situations to improve ecological validity and quantitative evaluation. In terms of setting up stress situations, scenarios that are closer to real competitions can be simulated to narrow the gap between the experimental situation and the actual competitive environment. At the same time, it is essential to introduce objective stress assessment tools to measure participants’ subjective stress perception, clarify the actual stress level borne by participants, and avoid result biases caused by the “disconnection between stress manipulation and perception.” Through the improvement of the above research design, the research results will be more practical.

## 5 Conclusion

This study explored the impact of mindfulness training on basketball players’ free throw performance in stressful situations through an 8-week mindfulness training intervention. It was found that mindfulness training can significantly improve basketball players’ mindfulness levels and mental toughness, and effectively enhance their free throw performance in stressful situations. In addition, there is a significant positive correlation between mindfulness, mental toughness, and the free-throw performance of basketball players. Therefore, mindfulness training is an effective psychological intervention method to improve basketball players’ free throw performance in stressful situations. This conclusion provides a scientific basis for the psychological training of basketball players and also offers a reference direction for psychological intervention research in other sports. It should be noted that this study has limitations such as a small sample size with weak representativeness, the existence of interfering factors in the experimental process, and the lack of ecological validity in the setting of stress situations.

## Data Availability

The raw data supporting the conclusions of this article will be made available by the authors, without undue reservation.
